# Retraction: Awareness and perception of malaria and dengue at school and college level in the district of Multan

**DOI:** 10.1371/journal.pone.0277592

**Published:** 2022-11-16

**Authors:** The PLOS ONE Editors

The *PLOS ONE* Editors retract this article [1] because it was identified as one of a series of submissions for which we have concerns about authorship, competing interests, and peer review. We regret that the issues were not addressed prior to the article’s publication.

All authors either did not respond directly or could not be reached.
